# Supplementary material for: In vivo study on the healing of bone defect treated with non-thermal atmospheric pressure gas discharge plasma
Source: PLoS One. 2021 Oct 11;16(10):e0255861. doi: 10.1371/journal.pone.0255861 (PMC8504758; doi:10.1371/journal.pone.0255861)
Supplement: Attachment — Submitted filename: comment.rtf [file pone.0255861.s001.rtf]

The submitted paper Shimatani et. all explain the use of plasmas for biomedical applications in encountering a growing interest, especially in the framework of In vivo study on the healing of bone defects, which aims at exploiting the action of low-power, atmospheric pressure plasmas for therapeutic purposes. Several applications have already reached the stage of clinical trials, while others are on their way, a large set of different plasma sources able to work at atmospheric pressure with low dissipated power has been created, and some of them are already certified as medical devices. From the scientific viewpoint, Overall, the manuscript reports on an interesting and well-written.  It is suitable for publication after clarification on the points raised below:
(1)  Please try to make uniforms, like  "non-thermal atmospheric pressure plasma (NTAPP)", "non-thermal atmospheric pressure gas discharge plasma", and "gas discharge plasma". Select uniform terminology.
(2) Overview of recent progress in applications of non-thermal plasma for therapeutic approaches has been developed to bone fracture healing and regeneration is need to be extended in the introduction.
(3)   Brief discussion about various RONS in the term of oxidation potential is needed in the introduction [ I refer https://doi.org/10.1016/j.cpme.2017.12.052, and https://doi.org/10.1016/j.cpme.2017.12.052 paper for a better explanation of plasma chemistry.]
(4)   Regarding the experimental setup, the authors claimed that, "The plasma jet assembly comprised a single electrode configuration. Thus, discharge currents passed through the biological target underneath the plasma."  If so then why don't you select DC power-driven plasma source? The application of AC-driven plasma sources doesn't support your statement.  Please supply some reasons/advantages of using AC have driven power source. For in vivo study, the floating jet is not electrically safe and it requires a much more breakdown voltage as compared to two electrode plasma jets. I think the logical explanation about why floating plasma jet is used in this experiment should be added. 
(5) This effect seen on bone is due to RONS or discharge current? Do you perform an experiment to support "discharge currents passed through the biological target underneath the plasma that will show a better bone healing effect"? If this statement is based on literature please cite the appropriate reference. Comparative study of dc- and ac-driven plasma source could support this statement.
(6) Current-voltage waveform and OES are greatly affected by target and without a target in this type of floating jet. Figure 1B, C, and D were measured with the target or without a target? Besides, please supply some reasons/advantages of using AC has driven power source. 
(7) Author assumed that highly reactive oxygen and nitrogen species (RONS) (e.g., hydroxyl radical OH), nitric oxide (NO), and oxygen-related neutral and positively and negatively charged species)could stimulate bone regeneration, for this why author selected helium gas? Can we expect a similar result in other economic working gas like argon, nitrogen, and air? 
(8) why there is only one positive current peak? 
At last, the Figures in the submitted manuscript are inverted. Please consider this during the submission of a revised manuscript.
